# Vestiges of the Natural History of Creation

**Published:** 1845-01

**Authors:** 


### Vestiges of the Natural History of Creation.
Octavo, pp. 390. Churchill, 1844.

This is a remarkable volume—small in compass—but embracing a wide range of inquiry, from worlds beyond the visible starry firmament, to the minutest structures of man and animals. The work is written with peculiar and classical terseness, reminding us very much of the style of Celsus. No name is prefixed—perhaps in order to avoid the snarls of the narrow-minded and bigotted saints of the present day, who will be up in arms against any man who presumes to think that this little globe we tread on, can number more than six thousand years since its first formation.

The first Chapter is on the Astral and Solar Systems. The following passage will convey some idea of our author's manner.

"The evidence of the existence of other astral systems besides our own is much more decided than might be expected, when we consider that the nearest of them must needs be placed at a mighty interval beyond our own. The elder Herschel, directing his wonderful tube towards the *sides* of our system, where stars are planted most rarely, and raising the powers of the instrument to the required pitch, was enabled with awe-struck mind to see suspended in the vast empyrean astral systems, or, as he called them, firmaments, resembling our own. Like light cloudlets to a certain power of the telescope, they resolved themselves, under a greater power, into stars, though these generally seemed no larger than the finest particles of diamond dust. The general forms of these systems are various; but one at least has been detected as bearing a striking resemblance to the supposed form of our own. The distances are also various, as proved by the different degrees of telescopic power necessary to bring them into view. The farthest observed by the astronomer were estimated by him as thirty-five thousand times more remote than Sirius, supposing its distance to be about twenty thousand millions of miles. It would thus appear, that not only does gravitation keep our earth in its place in the solar system, and the solar system in its place in our astral system, but it also may be presumed to have the mightier duty of preserving a local arrangement between that astral system and an immensity of others, through which the imagination is left to wander on and on without limit or stay, save that which is given by its inability to grasp the unbounded." 7.

Coming down to mother Earth, our author seems to think that all matter, constituting the Heavens and the Earth, was originally one mass—and that, previously to stellar and planetary formations, nebulous matter must have been a universal "fire-mist," of an immensely high temperature. A change in this heat must have led to agglomeration, and the various forms which we see around us, in the Solar and Stellar Systems. The solids, liquids, and gases of our Globe are reducible into fifty-five substances, called Elementary—six gases—forty-two metals—and several without names.

In the nebular hypothesis, satellites are considered as masses thrown off from their primaries, exactly as the primaries had been previously thrown off from the sun.

"The orbit of any satellite is also to be regarded as marking the bounds of the mass of the primary at the time when that satellite was thrown off; its speed likewise denotes the rapidity of the rotatory motion of the primary at that particular juncture. For example, the outermost of the four satellites of Jupiter revolves round his body at the distance of 1,180,582 miles, shewing that the planet was once 3,675,501 miles in circumference, instead of being, as now, only 89,170 miles in diameter. This large mass took rather more than sixteen days six hours and a half (the present revolutionary period of the outermost satellite) to rotate on its axis. The innermost satellite must have been formed when the planet was reduced to a circumference of 309,075 miles, and rotated in about forty-two hours and a half." 37.

By the same calculation the Earth, at a certain time after it was thrown off from the Sun, was no less than 482,000 miles in diameter—being sixty times more than at present. It then required 29½ days to perform its present diurnal rotation. At what period the Moon was thrown off from the Earth, it is impossible to form an estimate. It is evident, however, that our satellite is unprovided with an atmosphere—and highly probable that it is incapable of supporting animal life. It does not present any water on its surface; but its mountains and volcanos are on a stupendous scale. The Moon may be in progress to a formation that will exhibit seas and atmosphere, when it will present organic life like the Earth.

"We have seen reason to conclude that the primary condition of matter was that of a diffused mass, in which the component molecules were probably kept apart through the efficacy of heat; that portions of this agglomerated into suns, which threw off planets; that these planets were at first very much diffused, but gradually contracted by cooling to their present dimensions. Now, as to our own globe, there is a remarkable proof of its having been in a fluid state at the time when it was finally solidifying, in the fact of its being bulged at the equator, the very form which a soft revolving body takes, and must inevitably take, under the influence of centrifugal force. This bulging makes the equatorial exceed the polar diameter as 230 to 229, which has been demonstrated to be precisely the departure from a correct sphere which might be predicated from a knowledge of the amount of the mass and the rate of rotation." 41.

The *internal* heat of the Earth supports the above theory, as evinced by volcanos, thermal springs, &c.

*Era of the Primary Rocks.*—Dissections have gone far enough to shew that the basis rock of this globe is of a hard texture and crystalline constitution, of which granite may be considered the type. Over this, except in some mountainous projections, other rocks are disposed in strata, apparently deposited from water, but broken and disturbed by uneasy movements from beneath. Through these clefts may be, here and there, seen projections of rock of the primæval species—basalt—which had evidently been in a molten state at the time of eruption. The deposition of the aqueous, and the projection of the volcanic rocks have evidently taken place since the settlement of the Earth in its present form, and its agglomeration from the nebulous or vaporiform state. Such rocks unquestionably evince a combination of two or more of silica, mica, quartz, and hornblende, each of which is composed of a group of simple or elementary substances.

The deposition of Secondary Rocks or strata, is a process which is easily understood, being, in fact, still going on before our eyes, though on a comparatively small scale. In those days the seas were, in some places, more than a hundred miles in depth—and the submarine mountains of tremendous altitudes. The system of disintegration, under such circumstances, must have been enormous. The matters worn off, being carried into the neighbouring depths, and there deposited, became the components of the earliest stratified rocks—the first series of which (gneis and mica slate system) were of prodigious thickness—even one hundred miles! The seas, at that epoch, were probably of a boiling temperature. These early strata contain nothing but what are found in the primæval granite. They are the same in materials, but only changed in form. They exhibit no traces of vegetable or animal remains, that tell the wondrous tales of past history, in subsequent strata!

*Commencement of Organic Life.*—Here a new feature takes place in the stratified rocks—limestone—the carbonic acid gas of which plays such a part in animal and vegetable existence. Here, then, is an indication that vegetable and animal life began to appear—the carbonic acid gas being then, probably, too great in the atmosphere to support the life of land animals. And what are the organic remains discovered in this early stratum of earth? The unpretending forms of various Zoophites and Polypes, together with a few single and double-valved shell-fish—all of them creatures of the sea. Of vegetable remains none have come down to us of this period—perhaps they were too fragile to stand the wear and tear of the long journey.

Ascending to the next group of rocks—the Silurian—we find the traces of life become more abundant—the number of species extended—and important additions made in certain vestiges of fuci, or sea-plants, and fishes. From the lowest beds upwards, there are polypiarii, conchiferi, terebratula, mollusca, trilobate crustacea, &c.

*Red Sandstone.*—We now advance to a new Chapter in this wonderful history. This term has been applied to a series of strata, of enormous thickness, a mixture of flagstones, marly rocks, sandstones, in which is found bitumen, a remarkable new ingredient, being a vegetable production. Here we have the same forms of life continued, with the addition of fishes, some of which are of most extraordinary form, and shewing that the seas, in which the old red sandstone was deposited, must have swarmed with fishes. We can only notice one kind.

"The *pterichthys* has also strong bony plates over its body, arranged much like those of a tortoise, and has a long tail; but its most remarkable feature, and that which has suggested its name, is a pair of long and narrow wing-like appendages attached to the shoulders, which the creature is supposed to have erected for its defence when attacked by an enemy." 70.

Many of the fishes of that remote period were, in general outline, similar to fishes now existing, but they exhibited striking peculiarities in their internal organization. As yet there were no land animals—perhaps no dry land. The strata must have been first horizontal—and ultimately the granitic points must have been protruded above the level of the sea, forming our mountains—and that, most probably, by the action of volcanic fire.

"It may be called the *Era of the Oldest Mountains*, or, more boldly, of the formation of the detached portions of dry land over the hitherto watery surface of the globe—an important part of the designs of Providence, for which the time was now apparently come. It may be remarked, that volcanic disturbances and protrusions of trap took place throughout the whole period of the deposition of the primary rocks; but they were upon a comparatively limited scale, and probably all took place under water. It was only now that the central granitic masses of the great mountain ranges were thrown up, carrying up with them broken edges of the primary strata; a process which seems to have had this difference from the other, that it was the effect of a more tremendous force exerted at a lower depth in the earth, and generally acting in lines pervading a considerable portion of the earth's surface." 74.

*Land formed.*—This brings us to another great era in the history of our globe. Land was laid bare—rains came down, and formed into springs, rivers, and lakes. The secondary, or stratified rocks, consisting of a great and varied series, rest generally against the flanks of the upturned primary rocks—sometimes considerably inclined—at others, forming basin-like beds, nearly horizontal—in many places broken up and shifted by disturbances from below. There was now a theatre for the existence of land-plants and animals. In the lowest group of secondary rocks was the carboniferous or coal strata, of vegetable formation, and presenting numerous species and kinds of plants, many of which are now without descendants. The animal remains of this era are not numerous, in comparison with those which go before or succeed. Some of the fishes are of the sauroid character, partaking of the lizard nature, a genus of the Reptilia or land-class. Here, therefore, we have an approach to the air-breathing animals.

"Coal strata are nearly confined to the group termed the carboniferous formation. Thin beds are not unknown afterwards, but they occur only as a rare exception. It is therefore thought that the most important of the conditions which allowed of so abundant a terrestrial vegetation, had ceased about the time when this formation was closed. The high temperature was not one of the conditions which terminated, for there are evidences of it afterwards; but probably the superabundance of carbonic acid gas supposed to have existed during this era was expended before its close. There can be little doubt that the infusion of a large dose of this gas into the atmosphere at the present day would be attended by precisely the same circumstances as in the time of the carboniferous formation. Land animal life would not have a place on earth; vegetation would be enormous; and coal strata would be formed from the vast accumulations of woody matter, which would gather in every sea, near the mouths of great rivers." 91.

The termination of the carboniferous formation is marked by symptoms of volcanic violence—which some geologists have considered as the close of one system of things, and the beginning of another. Coal-beds generally lie in basins—but all such basins are broken up into pieces, some of which are tossed up on edge—others sunk, while numerous eruptions of volcanic rock (trap) are seen.

*Terrestrial Zoology.*—The new red sandstone presents the commencement of land animals, and is subdivided into several groups.

" The second group is a limestone with an infusion of magnesia. It is developed less generally than some others, but occurs conspicuously in England and Germany. Its place, above the red sandstone, shews the recurrence of circumstances favourable to animal life, and we accordingly find in it not only zoophytes, conchifera, and a few tribes of fish, but some faint traces of land plants, and a new and startling appearance—a reptile of saurian (lizard) character, analogous to the now existing family called monitors. Remains of this creature are found in cupriferous (copper-bearing) slate connected with the mountain limestone, at Mansfield and Glucksbrunn, in Germany, which may be taken as evidence that dry land existed in that age near those places." 95.

Various fishes of that era disappear entirely. The third group, chiefly sandstones, shew great agitation, and consequent diminution of animal life ; but present, for the first time, specimens of vertebrate animals of the sub-kingdom, namely, reptiles with imperfect respiratory apparatus. The specimens found are allied to the crocodile and lizard tribes of the present day—but stupendous in size.

" The animal to which the name *ichthyosaurus* has been given, was as long as a young whale, and it was fitted for living in the water, though breathing the atmosphere. It had the vertebral column and general bodily form of a fish, but to that were added the head and breast-bone of a lizard, and the paddles of the whale tribes. The beak, moreover, was that of a porpoise, and the teeth were those of a crocodile. It must have been a most destructive creature to the fish of those early seas." 97.

The pterodactyle was a lizard, furnished with wings to pursue its prey in the air. Crocodiles were 100 feet in length ! Marks of the feet of animals are discovered in the slabs of the new red sandstone. Some of these prints indicate small animals; but others denote birds of unusually large size.

*Oolite—Mammalia.*—The oolite is a limestone composed of small round grains, like eggs, or rather the roe of a fish. It is largely developed in England, France, and other countries, and is supposed to be of chemical formation. Organic remains are very numerous in the oolite system. The animals of the oolite are entirely different in species from those of the preceding age. The dry land of this period presented cycadeæ, a beautiful class of plants between the palms and conifers. The ocean at this time swelled with inhabitants. The polypiaria were so abundant as to form strata of themselves. Here was the BELEMNITE, with conical shell, air-chambers, and an ink-bag, with which it could muddle the water around it when pursued ! Land reptiles now abounded, and here, for the first time, we find remains of insects—also some fragments of the marsupial family, now the kangaroo.

*Cretaceous Formation.*—This, being a marine deposit, shews that the sea once flowed over the Cliffs of Dover, but the organic remains found in the chalk-beds, prove that dry land must have existed in the neighbourhood.

*Tertiary Formation—Mammalia.*—The chalk-beds are the highest strata that extend over a considerable space; but in hollows of these beds there have been formed series of strata—clays, limestones, marls, &c. to which the name of *tertiary formation* has been given.

"The hollows filled by the tertiary formation must be considered as the beds of estuaries left at the conclusion of the cretaceous period. We have seen that an estuary, either by the drifting up of its mouth, or a change of level in that quarter, may be supposed to have become an inland sheet of water, and that, by another change, of the reverse kind, it may be supposed to have become an estuary again. Such changes the Paris basin appears to have undergone oftener than once, for, first, we have there a fresh-water formation of clay and limestone beds; then, a marine-limestone formation; next, a second fresh-water formation, in which the material of the celebrated *plaster of Paris* (gypsum) is included; then, a second marine formation of sandy and limy beds; and finally, a third series of fresh-water strata. Such alternations occur in other examples of the tertiary formation likewise." 126.

Shells innumerable have been found in these tertiary deposits; but they sink into insignificance when compared with the mammalian remains discovered in the Parisian beds, and which prove that the land had now become the theatre of an extensive creation of the highest class of animals. Cuvier ascertained fifty species of these—all of them long since extinct. One was about the size of a horse, with lower jaw shorter than the upper —the feet presenting three large toes unprovided with claws. All these animals were herbivorous. One of them was 18 feet in length—had a mole-like form, capable of digging in the earth, and, as Dr. Buckland thinks, adapted both for land and water. In a subsequent period of the tertiary formation, the above animals disappear, and we have the elephant, hippopotamus, rhinoceros, &c. some of them startling from their size, as the mastodon twelve feet in height! The pliocene period of the tertiary deposits gives us the ox, the deer, camels, and other species of the *ruminantia.* Evidences of great volcanic disturbances are distinguishable in the tertiary deposits.

*Superficial Formations.*—Between the tertiary period, above alluded to, and the formation of man, there appears to have been an era of stiff blue clay, mingled with fragments of rock, of all sizes, travel-worn, to which geologists have given the name of *diluvium,* as being apparently the produce of some vast *flood,* or of the sea thrown into an unusual agitation. It seems then, when this stratum was laid down, much of the present dry land was under the ocean.

"The included masses of rock have been carefully inspected in many places, and traced to particular parent beds at considerable distances. Connected with these phenomena are certain rock surfaces on the slopes of hills and elsewhere, which exhibit groovings and scratchings, such as we might suppose would be produced by a quantity of loose blocks hurried along over them by a flood." 135.

This diluvium and these erratic blocks clearly indicate one last and long submersion of this globe's surface under the ocean.* But at length the land rose, or the sea subsided, as evidenced by the *terraces* which were successively the shores of the sea, and which are still seen, one above the other, on various coasts. They rise from twenty to twelve hundred feet above the level of the ocean.

"The irresistible inference from the phenomena is, that the highest was first the coast line; then an elevation took place, and the second highest became so, the first being now raised into the air and thrown inland. Then, upon another elevation, the sea began to form, at its new point of contact with the land, the third highest beach, and so on down to the platform nearest to the present sea-beach." 141.

This general submersion must, in all probability, have destroyed all terrestrial animal life; for no animals of anterior periods could be detected subsequently. The whole was changed—and a new creation took place when land re-appeared!

"Thus concludes the wondrous chapter of the earth's history which is told by geology. It takes up our globe at the period when its original incandescent state had nearly ceased; conducts it through what we have every reason to believe were vast, or at least very considerable, spaces of time, in the course of which many superficial changes took place, and vegetable and animal life was gradually developed; and drops it just at the point when man was apparently about to enter on the scene." 145.

Here our ingenious author enters into a long discussion as to whether the various tribes of animals were created and recreated by the direct personal power of the Almighty, or by the agency of *laws* which he laid down from the beginning. We do not see much interest in this dissertation; for either supposition infers the existence, the power, and the wisdom of the Creator.

"Those who would object to the hypothesis of a creation by the intervention of law, do not perhaps consider how powerful an argument in favour of the existence of God is lost by rejecting this doctrine. When all is seen to be the result of law, the idea of an Almighty Author becomes irresistible, for the creation of a law for an endless series of phenomena—an act of intelligence above all else that we can conceive—could have no other imaginable source, and tells, moreover, as powerfully for a sustaining as for an originating power." 158.

*Man.*—We have seen that, as soon as the temperature of the once boiling sea had settled down to a certain point, the ocean became tenanted by various kinds of animals, as evinced by their remains being found embedded in the strata formed beneath the waters. And when the land projected above the level of the ocean, and became tenantable, life was instantly produced, and series after series of animated creatures spread themselves over its surface. For how many millions and millions of years this production and reproductions of animals went on before man made his appearance on the scene no human being will ever know! In all probability, countless ages must have elapsed, before this master-piece of creation appeared. Our author's speculations on the how, the why, the

* The Barham rocks near Harrogate present remarkable specimens of the above.—*Rev.*

when and the wherefore this great event occured, will not give satisfaction to the present race of mankind! His hypothesis is three or four centuries in advance of the TIMES, and will be stigmatised by the modern saints as downright Atheism, although the author every where expresses his reverence for the consummate wisdom and power, and benevolence of the Supreme Being.

Our author evidently leans to the doctrine of "equivocal generation," considering the giving life to insects out of flint by Mr. Cross, as much a law of the Almighty, as the creation of the elephant or the homo.

"On the hypothesis here brought forward, the *acarus Crossii* was a type of being ordained from the beginning, and destined to be realized under certain physical conditions. When a human hand brought these conditions into the proper arrangement, it did an act akin to hundreds of familiar ones which we execute every day, and which are followed by natural results; but it did nothing more. The production of the insect, if it did take place as assumed, was as clearly an act of the Almighty himself, as if he had fashioned it with hands." 189.

Our author seems convinced that, instead of new creations at various epochs of the revolutions of this globe, one race reproduced its successor, according to the original laws of the Deity, and not by immediate interposition of God himself. This is the portion of his doctrine which will have to stand a hard test by the saints of the day!

"We have yet to advert to the most interesting class of facts connected with the laws of organic development. It is only in recent times that physiologists have observed that each animal passes, in the course of its germinal history, through a series of changes resembling the *permanent forms* of the various orders of animals inferior to it in the scale. Thus, for instance, an insect, standing at the head of the articulated animals, is, in the larva state, a true annelid, or worm, the annelida being the lowest in the same class. The embryo of a crab resembles the perfect animal of the inferior order myriapoda, and passes through all the forms of transition which characterize the intermediate tribes of crustacea. The frog, for some time after its birth, is a fish with external gills, and other organs fitting it for an aquatic life, all of which are changed as it advances to maturity, and becomes a land animal. The mammifer only passes through still more stages according to its higher place in the scale. Nor is man himself exempt from this law. His first form is that which is permanent in the animalcule. His organization gradually passes through conditions generally resembling a fish, a reptile, a bird, and the lower mammalia, before it attains its specific maturity. At one of the last stages of his fœtal career, he exhibits an intermaxillary bone, which is characteristic of the perfect ape: this is suppressed, and he may then be said to take leave of the simial type, and become a true human creature. Even, as we shall see, the varieties of his race are represented in the progressive development of an individual of the highest, before we see the adult Caucasian, the highest point yet attained in the animal scale." 199.

To come to particulars. The brain of man exceeds that of all other animals in complexity of organization and fulness of development. Yet, at one early period, it is only "a simple fold of nervous matter," with difficulty distinguishable into three parts. In this state it perfectly resembles the brain of an adult fish—thus assuming, *in transitu*, the form that, in the fish, is permanent.

"In a short time, however, the structure is become more complex, the parts more distinct, the spinal marrow better marked; it is now the brain of a reptile.

The change continues; by a singular motion, certain parts (*corpora quadragemina*) which had hitherto appeared on the upper surface, now pass towards the lower; the former is their permanent situation in fishes and reptiles, the latter in birds and mammalia. This is another advance in the scale, but more remains yet to be done. The complication of the organ increases; cavities termed *ventricles* are formed, which do not exist in fishes, reptiles, or birds; curiously organized parts, such as the corpora striata, are added; it is now the brain of the mammalia. Its last and final change alone seems wanting, that which shall render it the brain of MAN. And this change in time takes place.

"So also with the heart. This organ, in the mammalia, consists of four cavities, but in the reptiles of only three, and in fishes of two only, while in the articulated animals it is merely a prolonged tube. Now in the mammal fœtus, at a certain early stage, the organ has the form of a prolonged tube; and a human being may be said to have then the heart of an insect. Subsequently it is shortened and widened, and becomes divided by a contraction into two parts, a ventricle and an auricle; it is now the heart of a fish. A subdivision of the auricle afterwards makes a triple-chambered form, as in the heart of the reptile tribes; lastly, the ventricle being also subdivided, it becomes a full mammal heart." 201.

The tendency, says our author, of all these illustrations is to make us look to *development* as the principle which has been immediately concerned in the peopling of this Globe. We confess that, to us, it appears more philosophical to consider the first pair of each species of animated beings —man himself included—to have been created by the express command of GOD, and the races to have been perpetuated afterwards by the laws which we see daily operating before our eyes. Nor do we think that this view of the matter is at all deteriorated by the circumstance of its being consonant with Scripture. As for new kinds of creatures having been added from time to time, to the old stock, or brought on the stage as their successors, we consider them just as much original creations as their predecessors.

Our author seems to think it not impossible—perhaps not improbable— that man himself may, in some mysterious manner *develop* his physical and mental powers so as to produce a race as much superior to the present, as the present is to the beast of the field. From six to ten thousand years' experience of mankind does not give much sanction to this hope— for we fear that human nature, at the present day, is not a whit better than it was before the Flood.

After the masterly history of the human race by Dr. Pritchard, we need not enter on our author's views of this matter. He considers all the different races of mankind as sprung from one parent family.

Our author's opinions respecting the *mental distinctions* between man and animals may be easily gathered from the following passage.

"There is a general disinclination to regard mind in connexion with organization, from a fear that this must needs interfere with the cherished religious doctrine of the spirit of man, and lower him to the level of the brutes. A distinction is therefore drawn between our mental manifestations and those of the lower animals, the latter being comprehended under the term instinct, while ours are collectively described as mind, mind being again a received synonyme with soul, the immortal part of man. There is here a strange system of confusion and error, which it is most imprudent to regard as essential to religion, since candid investigations of nature tend to shew its untenableness. There is, in reality, nothing to prevent our regarding man as specially endowed with an immortal spirit, at the same time that his ordinary mental manifestations are looked upon as simple phenomena resulting from organization, those of the lower animals being phenomena absolutely the same in character, though developed within much narrower limits." 326.

In a chapter on the "Purpose and General Condition of the Animal Creation," we find many ingenious remarks, as well as important reflections, moral and religious. That *enjoyment* is the proper attendant of animal existence is proved by all observation and experience. But to form so vast a range of being, and to make it everywhere a source of gratification, seems beyond the power—or at all events the will—of the Creator!

"It appears at first difficult to reconcile with this idea the many miseries which we see all sentient beings, ourselves included, occasionally enduring. How, the sage has asked in every age, should a Being so transcendentally kind, have allowed of so large an admixture of evil in the condition of his creatures? Do we not at length find an answer to a certain extent satisfactory, in the view which has now been given of the constitution of nature? We there see the Deity operating in the most august of his works by fixed laws, an arrangement which, it is clear, only admits of the main and primary results being good, but disregards exceptions." 363.

Thus the winds, so useful to living creatures, will rise into hurricanes, destructive of animal and vegetable life. The love of exercise will sometimes lead to injuries that render individuals cripples for life. Even war, the most tremendous of evils, is caused by certain tendencies of human nature, as tenacity of supposed rights—resentment of injuries—desire of admiration—combativeness—love of excitement—ambition, and many other passions that are, within limits, indisputable benefits to us; but which, when carried beyond legitimate boundaries, inflict on us terrific sufferings! God has given us these tendencies for good purposes; but has not laid down any absolute obstruction to our misuse of them. He has, however, given us REASON to restrain our passions—and Religion to direct our steps in the paths of virtue. Still, there are evils for which we see no evident reason, and which the good and the bad experience alike.

"It is clear, moreover, from the whole scope of the natural laws, that the individual, as far as the present sphere of being is concerned, is to the Author of Nature a consideration of inferior moment. Everywhere we see the arrangements for the species perfect; the individual is left, as it were, to take his chance amidst the *mêlée* of the various laws affecting him. If he be found inferiorly endowed, or ill befalls him, there was at least no partiality against him. The system has the fairness of a lottery, in which every one has the like chance of drawing the prize.

"Yet it is also to be observed that few evils are altogether unmixed. God, contemplating apparently the unbending action of his great laws, has established others which appear to be designed to have a compensating, a repairing, and a consoling effect. Suppose, for instance, that from a defect in the power of development in a mother, her offspring is ushered into the world destitute of some of the most useful members, or blind, or deaf, or of imperfect intellect, there is ever to be found in the parents and other relatives, and in the surrounding public, a sympathy with the sufferer, which tends to make up for the deficiency, so that he is in the long run not much a loser. Indeed the benevolence implanted in our nature seeems to be an arrangement having for one of its principal objects to cause us, by sympathy and active aid, to remedy the evils unavoidably suffered by our fellow-creatures in the course of the operation of the other natural laws. And even in the sufferer himself, it is often found that a defect in one point is made up for by an extra power in another. The blind come to have a sense of touch much more acute than those who see. Persons born without hands have been known to acquire a power of using their feet for a number of the principal offices usually served by that member. I need hardly say how remarkably fatuity is compensated by the more than usual regard paid to the children born with it by their parents, and the zeal which others usually feel to protect and succour such persons. In short, we never see evil of any kind take place where there is not some remedy or compensating principle ready to interfere for its alleviation. And there can be no doubt that in this manner suffering of all kinds is very much relieved." 379.

We have dedicated a space to this remarkable work that may induce many of our readers to peruse the original. The author is, decidedly, a man of great information and reflection. He will have a host of saints in array against him, and many will join in the cry, from hypocrisy and self-interest. As we said before, his doctrines have come out a century before their time.

